# Grape seed pro-anthocyanidins ameliorates radiation-induced lung injury

**DOI:** 10.1111/jcmm.12276

**Published:** 2014-04-24

**Authors:** Yijuan Huang, Wen Liu, Hu Liu, Yanyong Yang, Jianguo Cui, Pei Zhang, Hainan Zhao, Feng He, Ying Cheng, Jin Ni, Jianming Cai, Bailong Li, Fu Gao

**Affiliations:** Department of Radiation Medicine, Faculty of Naval Medicine, Second Military Medical UniversityShanghai, China

**Keywords:** radiation-induced lung injury, grape seed pro-anthocyanidins, reactive oxygen species, epithelial–mesenchymal transition

## Abstract

Radiation-induced lung injury (RILI) is a potentially fatal and dose-limiting complication of thoracic radiotherapy. This study was to investigate the protective effects of grape seed pro-anthocyanidins (GSPs), an efficient antioxidant and anti-carcinogenic agent, on RILI. In our study, it was demonstrated that acute and late RILI was ameliorated after GSPs treatment possibly through suppressing TGF-β1/Smad3/Snail signalling pathway and modulating the levels of cytokines (interferon-γ, IL-4 and IL-13) derived from Th1/Th2 cells. In addition, a sustained high level of PGE2 was also maintained by GSPs treatment to limited fibroblast functions. As shown by electron spin resonance spectrometry, GSPs could scavenge hydroxyl radical (•OH) in a dose-dependent manner, which might account for the mitigation of lipid peroxidation and consequent apoptosis of lung cells. *In vitro*, GSPs radiosensitized lung cancer cell A549 while mitigating radiation injury on normal alveolar epithelial cell RLE-6TN. In conclusion, the results showed that GSPs protects mice from RILI through scavenging free radicals and modulating RILI-associated cytokines, suggesting GSPs as a novel protective agent in RILI.

## Introduction

Radiation-induced lung injury (RILI) is a disabling and potentially fatal, dose-limiting complication of thoracic radiotherapy for lung cancer, breast cancer, oesophageal cancer, lymphoma, thymoma [1] and total body irradiation [2]. The mechanisms involved in the initiation and perpetuation of RILI remain not well understood. Radiation-induced lung injury has classically been separated into two phases: pneumonitis and fibrosis. Doses of radiation (>8 Gy) to the chest can induce pneumonitis after approximately 2 months, while the fibrosis presents months to years later [3]. Clinically, RILI is characterized by interstitial infiltrates, progressive dyspnoea and worsening of pulmonary function that may lead to death from respiratory failure [4]. Radiation-induced lung injury was usually treated with steroids, which suffers from limited efficacy, serious side effects and the potential for fatal ‘recall’ pneumonitis when abruptly discontinued [5]. Despite the urgent requirements, there is remarkably little progress in the development of safe and effective therapeutic strategies.

Radiation-induced lung injury may arise as a result of the interaction of a complex cycle of chronic inflammation and altered expression of cytokines that causes production of reactive oxygen species (ROS) [6–9]. Thoracic irradiation perturbs both alveolar epithelium and vascular endothelium [10], resulting in loss of junctional integrity, increased permeability and physiological derangement [11]. Ionizing radiation exerts immediate biological effects largely through the generation of ROS such as hydroxyl radicals (•OH), which contributes 60–70% to ionizing radiation-induced injury by reacting rapidly with cellular macromolecules like DNA, lipids and proteins [12].

Radiation-induced lung injury is associated with increased secretions of inflammatory cytokines, chemokines, and inflammatory cells were also recruited into the lung parenchyma. Although it is difficult to grasp all of the complexities of the varied cell types and cytokine networks involved in lung fibrogenic responses, a large body of evidence converges on a single common theme: the central importance of the transforming growth factor-β1 (TGF-β1) signalling pathway [13]. The participation of several other pro-fibrogenic growth factors (PDGF, CTGF) and cytokines [IL-4, IL-13, interferon (IFN) -γ] is related to TGF-β1 function.

As a strong free radical scavenger, grape seed pro-anthocyanidins (GSPs) also exhibits anti-inflammatory, cardioprotective, immune modulating and anticarcinogenic activities [14,15]. Grape seed pro-anthocyanidins are also known as a sustained release antioxidant and can remain in the plasma and tissues for up to 7–10 days and exert antioxidant properties, which is mechanistically different from other water soluble antioxidants [16]. In addition, the LD50 of GSPs was greater than 5000 mg/kg of the bw, far more than treatment concentration, when administered orally *via* gastric intubation [16]. Given these properties of GSPs, it has great potential as a clinical therapeutic agent. In this study, we investigated the protective effects of GSPs on RILI and the underlying mechanism.

## Materials and methods

### Animals and GSPs treatments

All the protocols were approved by the Second Military Medical University, China, in accordance with the Guide for Care and Use of Laboratory Animals published by the US NIH (publication no. 96-01). Fibrosis-prone mice (8-week-old female C57BL/6J mice with an approximate bw of 20 g; Chinese Academy of Sciences, China) were used and randomly divided into three groups: non-irradiated control (*n* = 30); irradiation+saline (*n* = 30); and irradiation+GSPs (*n* = 30). The mice were housed three per cage and kept under standard laboratory conditions, during which sterilized food and water were supplied *ad libitum*. No animals had to be prematurely killed because of morbidity.

Grape seed pro-anthocyanidins was obtained from Pure-one Bio Technology Co. Ltd. (Shanghai, China). GSPs (30 mg/ml) was given by gavage about 1 hr before irradiation at the dose of 400 mg/kg and maintained through drinking (2 mg/ml) until 4 weeks after irradiation. Our measurements of GSPs consumption in the mice after irradiation indicated the GSPs intake was ∼8 mg/mouse/day.

### Cell culture and GSPs treatment

Rat alveolar type II cell line RLE-6TN (American Type Culture Collection) and human non-small cell lung cancer cell line A549 (American Type Culture Collection) were cultured in DMEM medium (10% foetal calf serum) at 37°C with 5% CO_2_ and 95% humidity. RLE-6TN cells and A549 cells were pre-treated with or without GSPs-containing PBS at 1 hr before irradiation and were further cultured for another 24 hrs and then switched to the normal DMEM medium. Cell viability and proliferation were determined by WST assay using a cell counting kit (Dojindo Laboratories, Kumamoto, Japan) and clonogenic assay as described previously [17].

### Irradiation

^60^Co source in the radiation centre (Faculty of Naval Medicine, Second Military Medical University, China) was used for irradiation. The mice received thoracic irradiation in a holder designed to immobilize anaesthetized mice so that only the lung volume was exposed to the beam. All radiated animals received a single dose of 15 Gy at a dose rate of 1 Gy/min. RLE-6TN and A549 were radiated with 8 Gy at a dose rate of 2 Gy/min.

### Lung extraction

At completion of experiments, mice were killed and the lung tissues were resected and weighed for calculating the lung coefficient (lung weight/bw). The lung lobes were then separated and the upper and lower lobes of right lung were used for western blot analysis and real-time quantitative RT-PCR respectively. The whole left lobe of the lung was used for histology and immunohistochemical analysis. We injected a volume (0.5–1.0 ml) of 4% paraformaldehyde solution into the left lobe of the lung to expand the alveoli and then placed the lobes in 4% paraformaldehyde solution for at least 48 hrs for fixation. The whole left lobe of lungs was embedded in paraffin and sections 5 μm thick were cut and placed on slides in preparation for staining.

### Histopathology and immunohistochemistry

Sections were used for terminal transferase mediated end labelling (TUNEL) and stained with haematoxylin and eosin, Masson's Trichrome, antibodies for TGF-β1 (1:200; Abcam, Cambridge, MA, USA), and p-Smad3 (1:200; Abcam), Snail (1:200; Abcam), Vimentin (1:200; Abcam), E-cadherin (1:200; Abcam), α-SMA (1:200; Abcam). Sections were analysed with the positive pixel algorithm in Aperio ImageScope (Aperio Technologies Inc.) as previously described [18]. The mean per cent positive pixels (stained) are presented.

### Western blot analysis

Frozen lung tissue was homogenized in M-PER (mammalian protein extraction reagent) containing a protein-stabilizing cocktail (Halt Protease Inhibitor Cocktail; Thermo Scientific, Beijing, China), 150 mM NaCl, and 1 mM EDTA. Lysates of RLE-6TN cells were prepared similarly. The lysates were mixed with an equal volume of sample buffer, denatured by boiling and then separated with 10% SDS-PAGE. The proteins were transferred to nitrocellulose membranes (Amersham, Arlington Heights, IL, USA), blocked with 10% milk and incubated overnight with E-cadherin (1:1500; Abcam), vimentin (1:1500; Abcam) and β-actin antibodies (1:1500; Abcam). The blots were then incubated with anti-rabbit IgG horseradish peroxidase conjugated antibodies (1:10,000; Abcam) for 1 hr at room temperature. Immunoreactivity on blots was detected with the LAS-4000 Luminescent Image Analyzer with CCD Camera (Fujifilm, Tokyo, Japan) and quantified by means of densitometry with Fuji Image Gauge software (version 4.0; Fujifilm).

### Cytokine and chemokine assays

Peripheral blood was collected after enucleation of eyeball and then centrifuged at 2520 × *g* and 4°C for 10 min. The serum was used for ELISA in accordance with the manufacturer's instructions.

### Real-time quantitative RT-PCR

Expression levels of RNA were quantitated by real-time PCR. We isolated total cellular RNA from lung tissue disrupted by Polytron in TRIzol reagent (Invitrogen, Carlsbad, CA, USA). SYBR Green assay and the ABI Prism 7900HT Sequence Detection System (Applied Biosystems, Rotkreuz, Switzerland) were used for the real-time quantitation of the RNA as previously described [19]. Primers used in this study were shown in Table 1.

**Table 1:** PCR Primers

Name	Forward	Reverse
TGF-β1	cttcagctccacagagaagaactgc	cacaatcatgttggacaactgctcc
ET-1	gctgcccaaagattctgaattctg	gatgatgtccaggtggcagaag
IFN-γ	actttgcttctgcctttcca	acaaggtcacccacaggaag
IL-4	cctgctcttctttctcgaatgt	tttcagtgatgtggacttggac
IL-13	cctggctcttgcttgcctt	ggtcttgtgtgatgttgctca
mPGES	gagttgaagtccaggccggctagccg	gctgaggaggcttcagctgctggtcac
GAPDH	ggtgcctgtcgttgtgttc	gctccttctggtgctgttg

### MDA assay

In the present study, levels of MDA were determined in lysates of radiated lung tissue by means of the MDA assay kit (Nanjing KeyGen Biotech. Co. Ltd., Nanjing, China), according to the manufacturer' instructions.

### Electron spin resonance spectrometry

We used 5,5-dimethyl-1-pyrroline N-oxide (DMPO; Labotec, Tokyo, Japan) to trap free radicals, especially •OH, and detected electron spin resonance (ESR) signals *via* an ESR spectrometer (EMX-8; Bruker BioSpin Corp, Berlin, Germany). As a standard of the reaction of •OH with DMPO, we produced •OH by means of the Fenton reaction with the mixture of 0.1% hydrogen peroxide and 0.1 mM ferrous chloride in the presence of 0.1 M DMPO and obtained spectra of the •DMPO-OH radicals at different concentrations of GSPs.

### Statistical analysis

Data are expressed as mean ± SEM for each experiment. Statistical analysis was performed by using one-way anova. Between groups, variance was determined using the Student-Newman-Keuls post-hoc test. *P* < 0.05 and *P* < 0.01 were considered as statistically significant.

## Results

### Protective effects of GSPs in a pre-clinical model of RILI

**Fig. 1:**
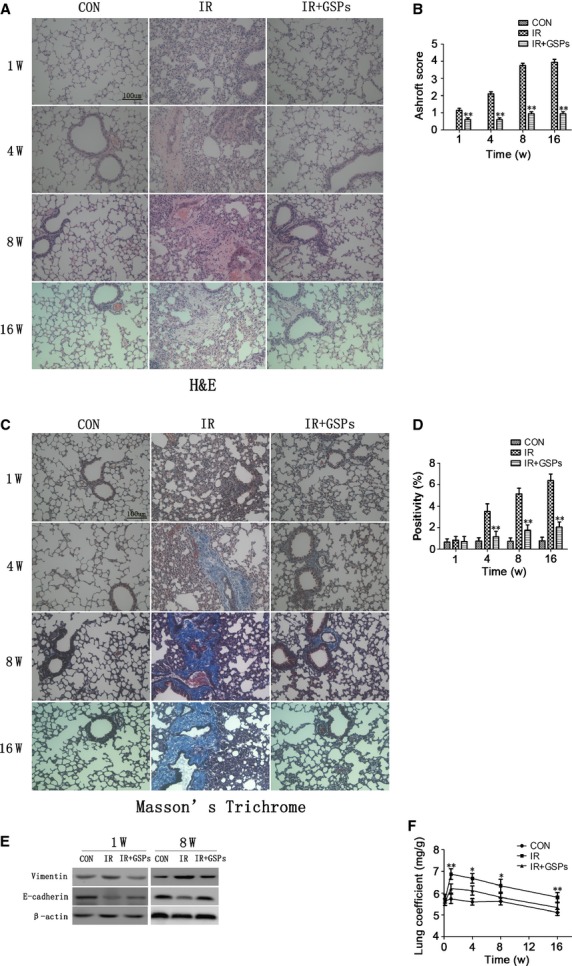
Protective effects of grape seed pro-anthocyanidins (GSPs) in a pre-clinical model of radiation-induced lung injury. (**A**) Photomicrographs of haematoxylin and eosin stained lung tissue sections from control mice, irradiated mice (15 Gy) and mice treated with GSPs isolated at 1, 4, 8 and 16 weeks after thoracic irradiation. Sections were analysed at ×200 magnification; bar 100 μm. (**B**) Ashcroft score. Semiquantitative histological analysis of pulmonary injury and fibrosis. (**C**) Representative images (×200) of Masson's Trichrome stain for collagen in lungs from mice taken at 1, 4, 8 and 16 weeks after irradiation; bar 100 μm. (**D**) Quantification of Masson's Trichrome stain for collagen content. (**E**) Western blot analysis of E-cadherin and vimentin expressions in the lung at 1 and 8 weeks after irradiation. (**F**) A bar graph of lung coefficient (lung weight/bw) from different groups. *P < 0.05 and **P < 0.01 *versus* the IR+GSPs groups (*n* = 6).

After a single 15 Gy irradiation, lungs showed markedly thickened alveolar walls, collapsed alveoli, diffuse accumulation of inflammatory cells and excessive deposition of extracellular matrix (ECM; Fig. 1A). Grape seed pro-anthocyanidins treatment significantly improved the pathological findings as indicated by the Ashcroft score (Fig. 1B) [20]. The amount of lung collagen visualized by Masson's trichrome staining after irradiation was also substantially decreased in GSPs-treated mice (Fig. 1C and D).

Next, we detected the role of GSPs in the transition of epithelial to mesenchymal that contributed to the formation of fibroblast and myofibroblast foci. Following irradiation, the protein level of epithelial maker E-cadherin deceased rapidly, but was significantly improved in GSPs-treated mice. In contrast, the level of a mesenchymal marker, vimentin, increased significantly after irradiation, while reduced in GSPs-treated mice (Fig. 1E).

In addition, lung coefficient (lung weight/bw) was used to evaluate the extent of pulmonary oedema. The lung coefficient increased significantly with a maximum at 1 week after irradiation and was significantly reduced in the GSPs-treated groups (Fig. 1F).

### GSPs modulated RILI-associated cytokines

**Fig. 2:**
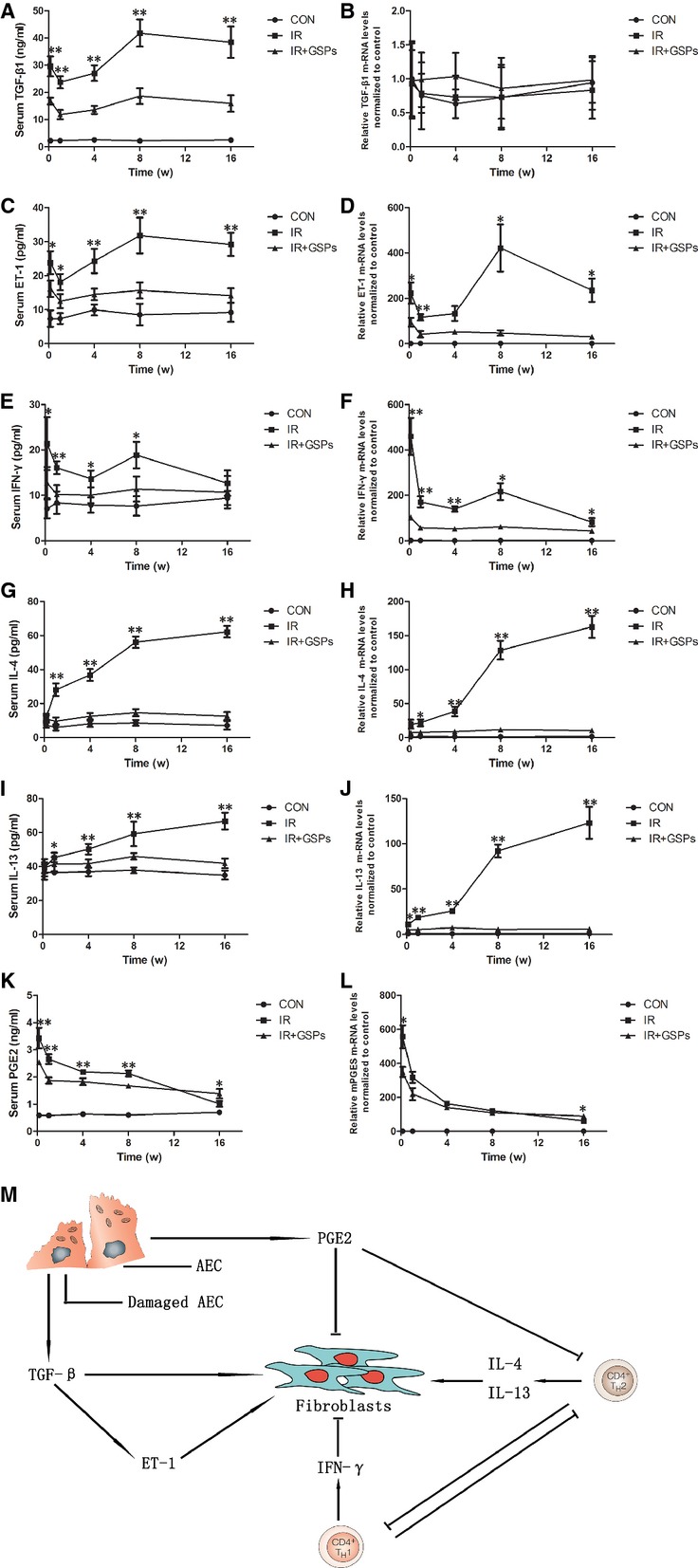
Grape seed pro-anthocyanidins (GSPs) modulated radiation-induced lung injury-associated cytokines. (**A**, **C**, **E**, **G**, **I**, **K**) TGF-β1, ET-1, IL-4, IL-13, IFN-γ and PGE2 levels in serum from mice taken at 1 day, 1, 4, 8 and 16 weeks after irradiation. (**B**, **D**, **F**, **H**, **J**, **L**) Relative TGF-β1, ET-1, IL-4, IL-13, IFN-γ and mPGES mRNA levels in lung from mice taken at 1 day, 1, 4, 8 and 16 weeks after irradiation. (**M**) TGF-β1, ET-1, IL-4, IL-13, IFN-γ and PGE2 act on fibroblasts. *P < 0.05 and **P < 0.01 *versus* IR+GSPs groups (*n* = 6).

Transforming growth factor-β1 is undoubtedly the most intensively studied regulator of the ECM, and production of TGF-β1 has been linked with the development of fibrosis in several diseases [21]. In this study, we confirmed that pulmonary irradiation led to remarkable increase in TGF-β1 that has been demonstrated two waves of increase occurred at 1 day and 8 weeks after irradiation (Fig. 2A). However, the increase in TGF-β1 in the level of mRNA was not detectable (Fig. 2B), indicating that the main level of control is not in the regulation of expression of the mRNA that encodes TGF-β1 but in the regulation of both the secretion and activation of latent TGF-β1. Interestingly, the increase in endothelin-1 (ET-1) manifested two waves not only in the level of protein, but also mRNA (Fig. 2C and D), indicating that modulation of its expression occurred mainly by transcriptional regulation of ET-1. However, the increase in the expression of both TGF-β1 and ET-1 was down-regulated in GSPs-treated mice.

Given that GSPs exhibited immune modulating properties [17], we hypothesized that GSPs regulated the balance of Th1/Th2 immune responses that played an important role in RILI. We found that pulmonary irradiation led to two waves of increase in IFN-γ (Fig. 2E) at the same trend as TGF-β1, while the level of Th2 cytokines IL-4 (Fig. 2G) and IL-13 (Fig. 2I) retained lower in the early stage, but stepped up progressively over the course of the experiment. These were further confirmed by the levels of mRNA measured in the lungs after pulmonary irradiation (Fig. 2F, H and J). Otherwise, GSPs treatment led to limitation of both Th1 and Th2 immune responses.

In addition, with the increase apoptosis of epithelial cells and conversion of fibroblasts to myofibroblasts after irradiation, there was a progressively impairment in the production of PGE2, while GSPs retained the high level of PGE2 even at the late stage after irradiation (Fig. 2K). This was confirmed by mRNA levels of microsomal prostaglandin E synthase (mPGES; Fig. 2L).

### GSPs suppressed TGF-β1/Smad3/Snail pathway

**Fig. 3:**
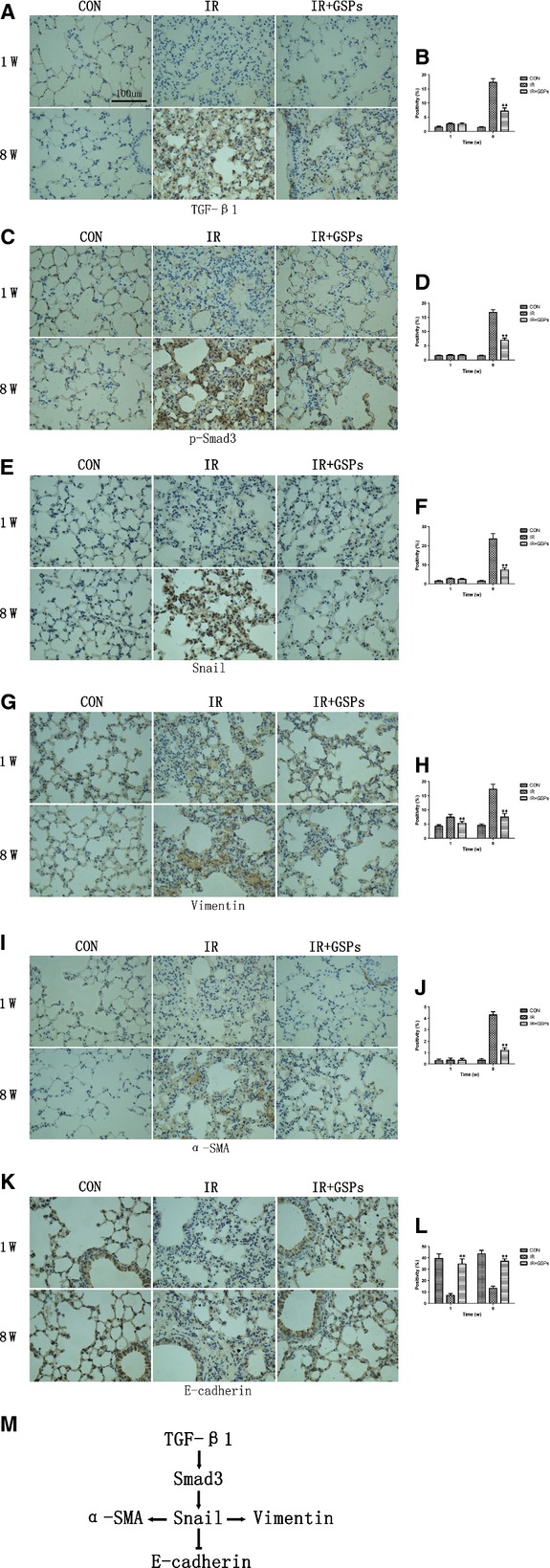
Grape seed pro-anthocyanidins (GSPs) suppressed TGF-β1/Smad3/Snail pathway. (**A**, **C**, **E**, **G**, **I**, **K**) Representative images of TGF-β1, p-Smad3, Snail, Vimentin, α-SMA and E-cadherin staining in lungs from mice killed at 1 and 8 weeks after irradiation. Sections were analysed at ×400 magnification; bar 100 μm. (**B**, **D**, **F**, **H**, **J**, **L**) Quantification of TGF-β1, p-Smad3, Snail, Vimentin, α-SMA and E-cadherin staining positivity. (**M**) Interrelationship among TGF-β1, p-Smad3, Snail, Vimentin, α-SMA and E-cadherin. *P < 0.05 and **P < 0.01 *versus* IR+GSPs groups (*n* = 6).

Given the predominant role of Smad3 and Snail in controlling the gene targets of TGF-β1 in fibroblasts [22,23], we immunostained TGF-β1, p-Smad3 and Snail at representative time-points 1 and 8 weeks after irradiation and found a significant inhibition by GSPs on TGF-β1, p-Smad3 and Snail at 8 weeks after irradiation (Fig. 3A–F), while no significant changes were observed at 1 week after irradiation. Consistent with these changes, the mesenchymal markers Vimentin and α-SMA were suppressed while the epithelial maker E-cadherin was protected by GSPs after irradiation (Fig. 3G–L).

### GSPs reduced the •OH level *in vitro* and attenuated oxidative damages and apoptosis in RILI

**Fig. 4:**
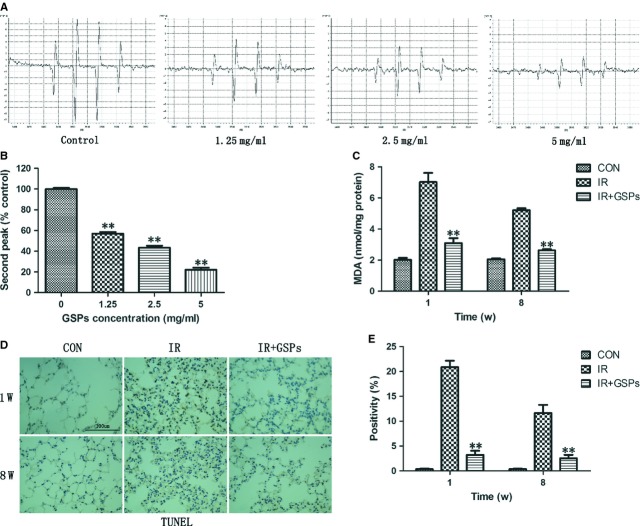
Grape seed pro-anthocyanidins (GSPs) reduced the •OH level *in vitro* and attenuated oxidative damages and apoptosis in radiation-induced lung injury. (**A**) •OH was produced by means of the Fenton reaction with the mixture of 0.1% hydrogen peroxide and 0.1 mM ferrous chloride in the presence of 0.1 M DMPO and obtained spectra of the •DMPO-OH radicals at different concentrations of GSPs by an electron spin resonance spectrometer. (**B**) Quantification of signals of the •DMPO-OH radical at the second peak normalized to control (*n* = 3). (**C**) Levels of MDA in lung tissues in different groups at 1 and 8 weeks after irradiation (*n* = 3). (**D**) Representative images (×400) of TUNEL staining in lungs from mice killed at 1 and 8 weeks after irradiation; bar 100 μm. (**E**) Quantification of TUNEL-positive cells. *P < 0.05 and **P < 0.01 *versus* IR+GSPs groups (*n* = 6).

To investigate the free radical scavenging ability of GSPs, we detected the signals of •DMPO-OH radical derived from the Fenton reaction in the presence of GSPs and found that GSPs reduced the •OH signals in a dose-dependent manner (Fig. 4A and B). These findings were corroborated by findings on MDA as alterations in lung tissues (Fig. 4C). In addition, apoptosis of lung cells induced by irradiation was reduced by GSPs as well (Fig. 4D and E).

### GSPs radiosensitized lung cancer cells A549 while mitigating radiation injury on normal alveolar epithelial cells RLE-6TN

**Fig. 5:**
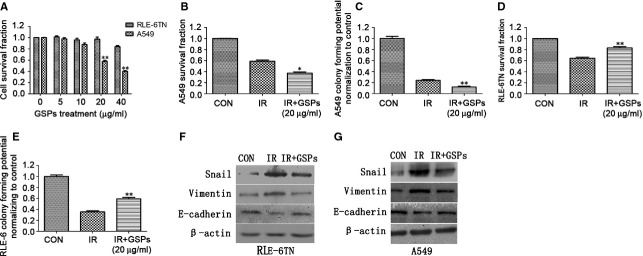
Grape seed pro-anthocyanidins (GSPs) radiosensitized lung cancer cells A549 while mitigating radiation injury on normal alveolar epithelial cells RLE-6TN. (**A**) The cytotoxicity of different concentrations of GSPs against A549 and RLE-6TN was determined by WST assay. Cell viability and proliferation of A549 (**B**, **C**) and RLE-6TN (**D**, **E**) cells in each group were determined by both WST assay and colonogenic assay. The cell viability and colony-forming potential of the cells were expressed in terms of per cent of control. (**F**) Epithelial to mesenchymal transition (EMT) regulator Snail and related markers E-cadherin and vimentin of RLE-6TN cells were determined at 96 hrs after irradiation. (**G**) EMT regulator Snail and related markers E-cadherin and vimentin of A549 cells were determined at 96 hrs after irradiation. *P < 0.05 and **P < 0.01 *versus* IR+GSPs groups (*n* = 6).

Grape seed pro-anthocyanidins inhibited cellular proliferation and diminished the viability of lung cancer cells A549 at a concentration 20 μg/ml where no toxicity was found on normal alveolar epithelial cells RLE-6TN (Fig. 5A). At this concentration, GSPs sensitized A549 to ionizing radiation (Fig. 5B and C) while mitigating radiation injury on RLE-6TN (Fig. 5D and E). In addition, epithelial to mesenchymal transition (EMT) of both RLE-6TN (Fig. 5F) and A549 (Fig. 5G) cells induced by radiation was attenuated by GSPs by measurement of the expression of E-cadherin and vimentin.

## Discussion

Radiation-induced lung injury is a chronic, progressive, irreversible and usually lethal lung disease resulting from thoracic radiation therapy, even with careful limitation of therapeutic radiation doses, for which there is no known effective therapy. In this study, we demonstrated that acute and late RILI was ameliorated after GSPs treatment possibly through suppressing TGF-β1/Smad3/Snail signalling pathway and modulating the levels of cytokines (IFN-γ, IL-4 and IL-13) derived from Th1/Th2 cells. It was also found that GSPs could scavenge hydroxyl radical (•OH) in a dose-dependent manner. In addition, GSPs radiosensitized lung cancer cell A549 while mitigating radiation injury on normal alveolar epithelial cell RLE-6TN.

Radiation-induced lung injury is a chronic inflammatory process, in which epithelial cell injury is the initial event that triggers a series of repair pathways that are in some way aberrant, leading to inappropriate fibrosis. The triggered epithelial cell becomes vulnerable to apoptosis, resulting in an apparent inability to restore the epithelial cell layer, accompanied by impaired regulation of myofibroblasts, which allowed fibrosis to proceed without constraint [24]. Following epithelial cells injury, there is accumulation of vascular exudates and inflammatory cells within the injured alveolar space. This exudate organizes with the influx and proliferation of fibroblasts and the emergence of the hallmark myofibroblast. Activated myofibroblasts, in turn, secrete angiotensinogen and hydrogen peroxide that induce alveolar epithelial cell apoptosis [25]. In this study, we showed that GSPs remarkably attenuated RILI probably through mitigating the excessive accumulation and activation of the main effector cell fibroblast. Fibroblast and myofibroblast foci are formed by proliferation of resident fibroblasts, migration of bone marrow-derived fibrocytes and transition of EMT. Our results showed that GSPs remarkably inhibited EMT *in vivo* and *in vitro*.

The results of epithelial injury are the release of several pro-inflammatory and pro-fibrotic mediators, including TGF-β1, PDGF, ET-1, TNF-α, *etc*. [26]. Although various cell types produce and respond to TGF-β, tissue fibrosis is mainly attributed to the TGF-β1 isoform, with circulating monocytes and tissue macrophages being the main cellular source [27]. In the bleomycin model of pulmonary fibrosis, alveolar macrophages are thought to produce nearly all of the active TGF-β1 [28]. Transforming growth factor-β1 is stored in the cell in an inactive form because of the association of the mature TGF-β1 peptide with the latency-associated peptide [29]. Liberation and activation of TGF-β1 are thus requisite steps to allow binding of the active TGF-β1 to TGF-β1 receptors (TGFBR2 and ALK5). This process leads to a series of signalling events through transmembrane receptors that phosphorylate the cytoplasmic TGF-β1 effectors, Smad2/3, which complexes with Smad4 and translocates to the nucleus, and modulate the transcription of target genes, including those encoding the ECM proteins pro-collagen-I and -III [30]. However, macrophage-derived TGF-β1 is often pro-fibrotic [31], whereas T cell-derived TGF-β1 seems to be suppressive [32]. Furthermore, a small amount of active TGF-β1 produced by αVβ6-expressing epithelium seems to suppress inflammation, while a larger amount is required to cause fibrosis [33]. These findings were confirmed by our results. We observed that C57BL/6J mice irradiated to the lungs demonstrated two waves of increase in cytokines (including TGF-β1, ET-1, IFN-γ and PGE2). Accordingly, the acute radiation-induced expression of suppressive TGF-β1 (small amount, possibly produced by αVβ6-expressing epithelium or T cells) was mildly affected by GSPs, whereas the fibrosis-related TGF-β1 (large amount, mainly derived from activated macrophages) expression at later time-points was significantly attenuated. In addition, p-Smad3 and Snail was attenuated simultaneously, indicating that GSPs performed anti-fibrotic property partially through inhibition of TGF-β1/Smad3/Snail signalling pathway and consequently attenuated EMT, characterized by lower expression of Vimentin and α-SMA and higher expression of E-cadherin.

Endothelin-1 is a potent endothelial-derived 21-amino-acid vasoconstrictor peptide [34] and one of the most potent regulators of ET-1 levels is TGF-β1 [35]. Transforming growth factor-β1 induces ET-1 expression preferentially through the ALK5/Smad3 pathway [36]. The ALK5/Smad3/ET-1 pathway leads to inhibition of endothelial cell migration and proliferation and is associated with a mature endothelium with increased expression of genes such as collagen type I or plasminogen activator inhibitor-1 (PAI-1). Recent studies have extended the role of ET-1 as a vasoconstrictor molecule to an essential modulator of several physiological processes such as the deposition of ECM components [37]. Endothelin-1 blockade has been shown to block TGF-β-induced myofibroblastic differentiation in cultured lung fibroblasts [38], and an ET-1 antagonist has been shown to delay death in a cohort of idiopathic pulmonary fibrosis patients [39]. In this study, we found that pulmonary irradiation led to increase in ET-1 at the same trend as TGF-β1 while was reduced by GSPs, indicating that GSPs contributed to the enhancement of neointimal hyperplasia and the decrease in matrix deposition in response to vascular injury.

Aberrant Th1/Th2 immune responses play an important role in the progression of RILI. Briefly, Th2 responses activate collagen deposition, whereas Th1 responses inhibit this process [21]. Th1 cytokines activate nitric-oxide synthase 2 in anti-fibrotic M1 macrophages, whereas Th2 cytokines IL-4 and IL-13 preferentially stimulate arginase-1 activity in pro-fibrotic M2 macrophages [40,41]. Each of the two main Th2 cytokines has a distinct role in the regulation of tissue remodelling and fibrosis. IL-4 and IL-13 can function separately or together with the TGF-β1/Smad3 signalling pathway to stimulate the collagen-producing machinery [42,43]. Interferon-γ is a Th1-derived cytokine with the properties that exert anti-fibrotic and immunomodulatory effects [21]. Interferon-γ suppresses fibrosis at least in part by inhibiting signalling by the major pro-fibrotic factors IL-4, IL-13 and TGF-β1 [44]. As a major effector cytokine of Th1 immunity, IFN-γ auto-amplifies Th1 responses and cross-inhibits differentiation and function of Th2 cells and expression of Th2-derived cytokines. In addition, IFN-γ induces expression of Smad7, an inhibitory Smad family member, and thus inhibits TGF-β-induced activation of Smad3 [45]. STAT1 activated by IFN-γ also directly binds Smad3 and inhibits its function [46]. Our results showed that pulmonary irradiation led to immediate in IFN-γ, indicating that Th1 cells might participate in the early inflammatory responses to irradiation. While the level of Th2 cytokines IL-4 and IL-13 was low in the early stage, but stepped up gradually and kept at a high level till the end of the observation. These were consistent with the development of fibrosis. In addition, we found the expression of cytokines sharply increased at 8 weeks after pulmonary irradiation as also reported in other studies [6]. This was associated with increases in oxidative damage to DNA, hypoxia, decreased lung perfusion and increased TGF-β1 expression. However, GSPs treatment led to limited Th1 and Th2 immune responses after irradiation, possibly because of the remarkable antioxidant property of GSPs that drastically attenuated tissue damage that attracted and activated massive inflammatory cells. The extent of the inflammatory response and of radiation-induced fibrosis was reduced by GSPs, suggesting that the reductions in the inflammatory process during the pneumonitis may lead to a partial short circuiting of the chronic cyclic inflammatory response, although several experimental models show that the amount of fibrosis is not necessarily linked with the severity of inflammation [21]. However, the potential contributions of GSPs to the processes involved in immunoregulation and the exact underlying mechanism will be the focus of future studies.

Prostaglandin E2 (PGE2), derived from arachidonic acid through the action of cyclooxygenase, is commonly considered a potent pro-inflammatory mediator and is involved in several inflammatory diseases. In the lung, PGE2 has a role in limiting the immune-inflammatory response as well as tissue repair processes [47]. Prostaglandin E2 is involved in the regulation of lymphocyte trafficking into tissue and also inhibits Th2 differentiation. In the present study, pulmonary irradiation stimulated the production of several cytokines in the early stage and consequently stimulated the release of great amounts of PGE2 by fibroblast. PGE2, in turn, exerted a negative feedback on cytokine production and down-regulate fibroblast metabolic functions including proliferation, collagen synthesis and transition to myofibroblasts [48]. In the later stage, there was an impairment in the production of PGE2, as previously observed in fibrotic fibroblasts and damaged epithelial cells [47,49], thus potentially leading to the persistence of immune activation and chronic inflammation. However, GSPs protected epithelial cells against irradiation, thus ensuring higher level of PGE2 to control fibroblast functions in the later stage, indicating that GSPs attenuated RILI partially because of epithelium protection.

Reactive oxygen species plays a critical role in the pathogenesis of RILI. There are at least three potential sources of ROS within the irradiated lung, first that which is produced as a direct result of radiation, second that which is generated by inflammatory cells [50] and third that from damaged mitochondria because of leakage from the electron transport chain [51]. Our results demonstrated that GSPs exhibited significant free radical scavenging ability in a cell-free Fenton system. These may account for the mechanism in the mitigation of RILI. Reduction in ROS led to protection of epithelium and endothelium and consequently to reduce fibroblast recruitment and vascular leak, which may be excessive when injury leads to fibrosis rather than to repair.

Finally, whenever any protective agent is given in combination with radiation therapy there is a concern that it may protect tumour as well. Consequently, this study also investigated the effect of GSPs on human non-small cell lung cancer cell line A549. We found that GSPs manifested tumour killing activity on A549 and a trend towards tumour sensitization even at low concentrations. This is consistent with other reports that GSPs has shown potential as an anti-cancer agent in several different cancer cell types [16]. The property that GSPs radiosensitize lung cancer cells while mitigating normal tissue injury could be of benefit for lung cancer patients to enhance the therapeutic efficacy by simultaneously reducing the RILI.

In conclusion, we found that GSPs protected mice from RILI, reduced the radiation damage on lung structure and regulated the imbalance of Th1/Th2-related cytokines. Reactive oxygen species level was also reduced by GSPs treatment as well as the apoptosis and lipid oxidation level. Finally, GSPs was found to sensitize tumour cells to ionizing radiation while exert protective roles on normal lung epithelial cells. This study suggests GSPs as a safe and effective agent against RILI, indicating great potential in clinical application.
